# Comparison of gauze packing, sponge-based, and hemostatic surgicel wound stasis dressings to treat hemorrhages from grade IV liver injuries: An experimental study

**DOI:** 10.1016/j.heliyon.2024.e39894

**Published:** 2024-10-26

**Authors:** Mohammad Mehdi Lashkarizadeh, Arshin Ghaedi, Hojat Abolghasemi, Mina Rabiee, Davood Mehrabani, Samad Ahadian, Aida Bazrgar, Shaqayeq Moqbel Esfahani, Shahram Paydar

**Affiliations:** a Shiraz Organ Transplant Center, Shiraz University of Medical Sciences, Shiraz, Iran; b Trauma Research Center, Shahid Rajaee (Emtiaz) Trauma Hospital, Shiraz University of Medical Sciences, Shiraz, Iran; c Student Research Committee, School of Medicine, Shiraz University of Medical Sciences, Shiraz, Iran; d School of Medicine, Tehran University of Medical Sciences (TUMS), Division of Hepatopancreatobiliary & Liver Transplantation, Imam Khomeini Hospital Complex, Iran; e Stem Cell Technology Research Center, Shiraz University of Medical Sciences, Shiraz, Iran; f Department of Genetics, Islamic Azad University, Shahre-Kord Branch, Share-Kord, Iran

**Keywords:** Trauma, Bleeding, Hemorrhage, Liver, Surgicel, XStat

## Abstract

**Background:**

Uncontrolled bleeding is still the major factor leading to preventable deaths following trauma. This study sought to assess the effectiveness of mini sponge-based wound stasis, cellulose-based local hemostatic, and traditional gauze dressings for the control of hemorrhages resulting from grade 4 liver injuries in rats.

**Methods:**

Thirty Sprague-Dawley rats were divided into three equal groups. In the first group, a liver laceration was treated with gauze packing. The second group received XStat minisponge dressing (MDS), and the third group was administered a combination of MDS dressing and Surgicel hemostatic agent. After gaining access to the intra-abdominal cavity, a liver laceration measuring 10 mm in length, 5 mm in depth, and extending to the middle lobe was created. The dressings were removed after 2 and 10 min to assess the amount of bleeding, and any bleeding was documented again after 48 h. Intraperitoneal adhesions were evaluated during euthanasia.

**Results:**

At 2 min post-injury, the gauze packing group had an average bleeding volume of 0.97 ± 0.15 mL, compared to 1.08 ± 0.25 mL in the MDS group (P = 0.26) and 1.02 ± 0.18 mL in the MDS + Surgicel group (P = 0.69). At 10 min, the bleeding volumes were 0.13 ± 0.05 mL, 0.22 ± 0.01 mL (P = 0.09), and 0.14 ± 0.05 mL (P = 0.19), respectively. At 48 h, significant differences were observed in bleeding volumes (gauze: 0.55 ± 0.18 mL, MDS: 1.15 ± 0.21 mL, MDS + Surgicel: 0.82 ± 0.06 mL, P < 0.001). Mortality rates after 14 days were 0 % in the gauze packing group, 60 % in the MDS group (P = 0.001), and 60 % in the MDS + Surgicel group (P = 0.001). The gauze packing group displayed no adhesions, while the other groups exhibited adhesions in the liver, bowel, omentum, and abdominal wall.

**Conclusion:**

Our results indicate that, when it comes to managing bleeding in severe liver injuries, traditional gauze packing remains the tried-and-true method, showing superior effectiveness in terms of blood loss and mortality when compared to XStat minisponge dressings and the fibrillar Surgicel hemostatic agents.

## 1 Introduction

The National Trauma Institute estimates that trauma is the leading cause of mortality for Americans between the ages of 1 and 46, with annual healthcare costs of $670 billion [1]. Abdominal trauma, affecting less than 10 % of injury cases but up to a third of severe injuries, predominantly impacts organs like the liver, kidney, and spleen due to their vulnerability [[2], [3], [4], [5]]. Uncontrolled bleeding stands as the primary preventable factor contributing to fatalities following abdominal trauma [6]. Liver injuries, particularly severe ones involving large parenchymal ruptures or deep lacerations, pose significant mortality risks primarily due to uncontrolled hemorrhage [7]. In general, surgical treatments, blood loss management, and pharmaceutical medications are employed to control hepatic hemorrhage [[8], [9], [10], [11]]. A local surgical intervention would be the highest priority in these patients with ongoing bleeding [11,12]. Managing ongoing bleeding caused by liver injuries with surgical methods may be difficult because the hepatic parenchyma cannot be readily repaired using sutures or packing. This complication is exacerbated in seriously damaged individuals who have extensive coagulation disorders. Furthermore, the complicated sinusoidal architecture of the liver offers a considerable barrier to efficient hemostasis inside hepatic tissue [13].

Despite improvements in surgical techniques, one of the difficulties surgeons may have in preserving patients' lives is limiting bleeding from liver parenchymal tissue [14,15]. In addition to the significant blood loss, the high mortality and morbidity rates may also be related to the prolonged time required to stop the bleeding [16]. As a result, various studies have investigated hemostatic techniques in liver procedures [17,18]. Hemostatic agents have been extensively studied in both experimental and clinical settings. Traditional methods such as gauze packing have been widely used due to their simplicity and effectiveness in controlling bleeding [19]. For instance, Peri-hepatic gauze packing (PHGP) is a widely used procedure for controlling bleeding after severe liver injuries [20]. However, the development of advanced hemostatic agents, such as the XStat™ minisponge dressing (MSD), Surgicel and hydrogel-based dressings, has provided new options for managing hemorrhage, especially in complex or non-compressible wounds [[21], [22], [23], [24], [25]]. XStat, originally developed for military use, has shown promising results in controlling severe hemorrhage in combat settings [21]. Studies by Mueller et al. compared XStat to combat gauze and demonstrated its effectiveness in rapidly controlling arterial bleeding [26]. MSD is also authorized for non-compressible wound treatment in battlefield/trauma scenarios. This involves the utilization of densely compressed medical sponges that can effectively stop high-flow arterial bleeding within a few seconds [27]. However, these studies primarily focused on external wounds, leaving a gap in knowledge regarding its efficacy for internal injuries.

Surgicel, a cellulose-based hemostatic agent, has been used in various surgical procedures due to its ability to facilitate clot formation and achieve hemostasis [22]. Clinical studies have reported its effectiveness in reducing intraoperative bleeding and improving surgical outcomes [28,29].

Traditional gauze packing has long been employed as a standard method to control hemorrhage, but advancements in hemostatic technologies have introduced alternatives such as sponge-based dressings and cellulose-based agents like Surgicel. Despite these advancements, there is still an ongoing debate about the most effective approach to manage severe hepatic bleeding. This study aims to compare the efficacy of three different types of dressings—traditional gauze packing, mini sponge-based wound stasis (XStat), and a combination of mini sponge-based dressings with Surgicel—specifically in the context of grade IV liver injuries. By utilizing a controlled rat model, we seek to evaluate the hemostatic effectiveness, mortality rates, and postoperative complications associated with each treatment method. Understanding the relative benefits and limitations of these wound stasis techniques is crucial for guiding clinical decisions in trauma care settings. This research endeavors to provide comprehensive data on the performance of these dressings, thereby contributing to the optimization of hemorrhage control strategies in cases of severe liver trauma.

## 2 Materials and methods

This experimental study was approved by the Animal Ethics Committee of Shiraz University of Medical Sciences, Shiraz, Iran (Ethical code number: IR.SUMS.MED.REC.1398.143). Moreover, this study complied with the International Helsinki Convention's animal experimentation agreement.

**Fig. 1:**
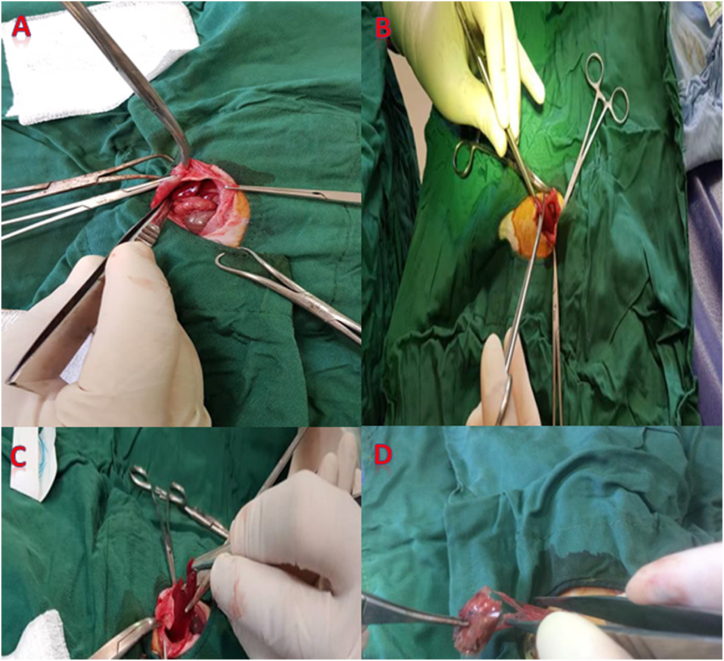
MDS vs. gauze packing. A: Exposure of organs after laparotomy. B: Induction of hepatic injury. C: Packing of liver and abdominal closure. D: Removal of the packing in a second laparotomy.

**Fig. 2:**
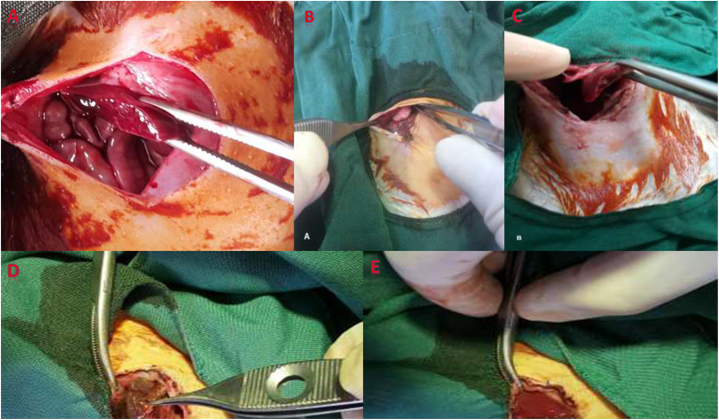
MDS + surgicel vs. gauze packing. **A:** Middle lobe liver laceration model. **B:** Second look of perihepatic gauze packing with a barrier between packing and tissues, 48 h after injury. **C:** Gauze removal with complete liver hemostasis without adhesion formation. **D:** Adhesion in MDS + surgicel group to Omentum. **E:** Adhesion in MDS + surgicel group to liver.

A total of 30 adult male Sprague-Dawley rats with a mean weight of 250 ± 25 g were purchased from the animal house at the Shiraz University of Medical Sciences in Shiraz, Iran, and kept in isolation for 7 days before the experiment under conditions that included a constant temperature of 22 °C, a humidity of 55 %, and 12-h cycles of light and dark. Food was denied the night before the experiments, but the animals were allowed free access to water. Rats were divided into three equal groups of 10 each. In the first group, liver lacerations were done and gauze packing was used. XStatTM minisponge dressing (MSD, China) was used for the second group, and the third group received a combination of MDS dressing and Surgicel® absorbable fibrillar hemostatic agent (Johnson & Johnson, USA). The animals underwent anesthesia through intramuscular injection of ketamine (50 mg/kg; Alfasan International, Woerden, Netherlands) and xylazine (10 mg/kg; Alfasan International, Woerden, Netherlands). To access the intra-abdominal cavity, a midline abdominal laparotomy was conducted. Within this cavity, a standardized liver laceration measuring 10 mm in length, 5 mm in depth, and width was created on the middle lobe of the liver using a scalpel blade. This procedure occurred following anesthesia administration and sterilization of the surgical area (refer to Fig. 1, Fig. 2). To ensure consistency and minimize procedural bias, all steps were carried out by a single surgeon.

After 2 min of uncontrollable bleeding, free blood in the peritoneal cavity was collected and quantified using a syringe, and the liver was packed once again. The packs were taken out after 10 min to determine how much blood had been lost. In all groups, a sterilized nylon was used as a barrier between the dressing and the liver and bowel to prevent intraperitoneal adhesion development (Fig. 1, Fig. 2).

The animals returned to their cages, where a veterinarian looked after them. The animals were brought back to the operating room 48 h after the original injury to remove the packing, calculate any free blood, and ultimately close the fascia once again. Wherever blood was still flowing after the gauze was removed, cauterization was performed. After the first operation, 14 days later, euthanasia was conducted, and during necropsy and packing removal, the amount of free intraperitoneal blood and adhesions were measured (Fig. 1, Fig. 2).

We decided to only use male rats in this study for a number of well-studied scientific reasons that are consistent with the goals and plan of our research. According to the following arguments, such as limiting variability and resource limitations, it was decided to concentrate on male animals. We recognize that excluding female rats may restrict the generalizability of our results to both sexes. However, we feel that this preliminary study serves as an important stepping stone for future examination, and we want to expand our research to include female individuals in upcoming studies to give a more thorough explanation of the impacts identified.

### 2.1 Statistical analysis

The results were presented as mean ± standard deviation (SD). The Fisher's Exact and Mann-Whitney U-tests were used for statistical comparisons (SPSS Statistics software, version 22, Chicago, IL). Statistics were considered significant with P-values less than 0.05. Also, the authors used ChatGPT-3.5 (OpenAI) in order to improve readability and language of some parts of the study.

## 3 Results

### 3.1 Bleeding assessment

Regarding intra-peritoneal bleeding, no statistical difference was observed between the gauze packing and MDS group at 2 min after liver injury [MDS vs. gauze packing: 1.08 ± 0.25 mL vs. 0.97 ± 0.15 mL, (P = 0.26)] or at 10 min after liver injury [MDS vs. gauze packing: 0.22 ± 0.01 mL vs. 0.13 ± 0.05 mL, (P = 0.09)]. However, there was a significant difference in the amount of intra-abdominal hemorrhage at 48 h of explorative laparotomy [MDS vs. gauze packing: 1.15 ± 0.21 mL vs. 0.55 ± 0.18 mL, P < 0.001)]. After 48 h, none of them needed cauterization or repacking.

No statistical difference was noted for intra-peritoneal bleeding between the gauze packing and MDS + surgicel fibrillar hemostat group at 2 min after liver injury [MDS + surgicel vs. gauze packing: 1.02 ± 0.18 mL vs. 0.97 ± 0.15 mL, (P = 0.69)] or at 10 min after liver injury [MDS + surgicel vs. gauze packing: 0.14 ± 0.05 mL vs. 0.13 ± 0.05 mL, (P = 0.19)]. However, there was a significant difference in the amount of intra-abdominal hemorrhage at 48 h of explorative laparotomy [MDS + surgicel vs. gauze packing: 0.82 ± 0.06 mL vs. 0.55 ± 0.18 mL, P < 0.001)]. None of them needed cauterization and repacking after 48-h interval (Table 1).

**Table 1:** Mean amount of shed blood at 2 min, 10 min & 48 h after liver injury.

Group	Simple Gauze Packing Group	Bleeding Stop + Surgicel Packing Group	P-value Simple Gauze P-value BS-Surgicel
Mean ± SD amount of shed blood at 2 min (ml)	0.97 ± 0.15	1.02 ± 0.18	0.19
Mean ± SD amount of shed blood at 10 min (ml)	0.13 ± 0.05	0.14 ± 0.052	0.098
Mean ± SD amount of shed blood at 48 h (ml)	0.55 ± 0.184	0.82 ± 0.063	0.0001

### 3.2 Mortality assessment

**Fig. 3:**
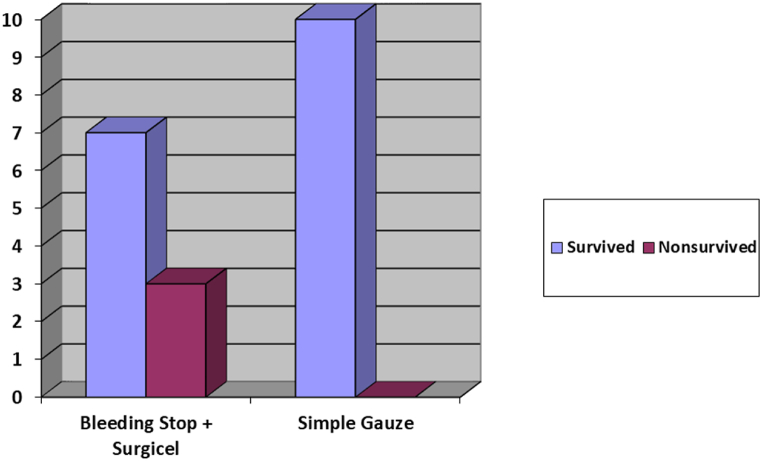
Mortality after 14 days in both group.

Compared to the gauze packing group, the MDS group illustrated a higher mortality rate after 14 days (60 % vs. 0 %, P = 0.001). The mortality rate during the first 48 h was 50 %, and 50 % of mortalities happened the next days. When the MDS + surgicel group was compared to the gauze packing group, a higher mortality rate was demonstrated in the MDS + surgicel group after 14 days (60 % vs. 0 %, P = 0.001). The mortality rate was 20 % during the first 48 h, and only one death was noticed the days after (Fig. 3).

### 3.3 Intraperitoneal adhesions

Regarding intraperitoneal adhesions and comparison of the MDS group with the gauze packing group, the adhesions were visible in 4 cases of the MDS group; even nylon barrier was present between MDS and the bowel. No adhesion was noted after placing the nylon barrier in the gauze packing group. In the MDS group, phlegmon formation occurred in two cases, and adhesion was seen in the liver, omentum, bowel, and abdominal wall, respectively.

Comparing MDS + surgicel group with gauze packing group, intraperitoneal adhesions were visible in 4 cases of the MDS + surgicel group, despite the nylon barrier between MDS + surgicel and the bowel, but in the gauze packing group, no adhesions were noted even the nylon barrier existed. MDS + surgicel resulted in adhesions in the liver, omentum, bowel, and abdominal wall in 5, 4, 3, and 1 cases, respectively; while adhering to the omentum and bowel was difficult to remove the packs.

## 4 Discussion

Uncontrolled bleeding is the leading cause of avoidable deaths after trauma, frequently from an unreachable internal source that cannot be compressed [30,31]. Local surgical management remains the foremost focus in patients with continuous truncal bleeding. Local surgical control is complicated due to the parenchymal nature of some organs being less amenable for suturing or simple coagulation methods by a surgical device. Because of the negative consequences of perioperative blood loss and blood transfusion in patients undergoing liver surgery, researchers actively seek novel and efficient approaches to reduce blood loss and the need for blood product transfusions, especially in cases of compromised hemostasis [32].

In fact, substantial blood loss and the time required to stop the bleeding might result in several difficulties for liver surgery [33]. Because most topical hemostatic medications used to control liver bleeding rely on hepatic homeostatic function, most of the topical hemostatic treatments are ineffective in controlling liver bleeding in individuals with critical physiologic states. As a result, researchers have focused on hemostatic drugs that are not reliant on the regular functioning of the hemostatic system [32].

Numerous local hemostatic agents have been introduced to manage localized hemorrhages. In preclinical assessments focusing on external bleeding, several hemostatic agents have effectively reduced blood loss and improved patient survival [34,35]. Commercially, they are offered in both military and civilian healthcare facilities [27,36]. However, for internal use, only off-label and uncontrolled experiences of refractory bleeding have been documented [27], while their safety and efficacy still need more investigations. A recent study conducted at the US Army Institute for Surgical Research has underscored the imperative for such investigations, particularly about a product known as WoundStat, which has the potential for systemic circulation-based embolization [34].

The XStat™ dressing is a mini sponge liquid-expandable bleeding stop product comprised of chitosan covered with a hydrogel [32]. Chitosan, a complex carbohydrate, is biodegradable and non-toxic but lacks intrinsic hemostatic properties. Yet, it can activate the body's natural coagulation system, stimulating the cascade of clot formation [37,38]. Additionally, it has the potential to absorb water, leading to a heightened concentration of clotting elements at the precise site of bleeding [39]; nevertheless, it may deteriorate the condition and exacerbate the bleeding in patients with coagulopathies or any abnormality in liver function via continuous sucking of the wound [40]. Therefore, in these patients, the packing pressure is more effective than the gauze packing method [41,42], which can also explain similar findings of our study.

In another study, a chitosan-based dressing was evaluated in a high-grade liver injury [42], demonstrating that a 90-min follow-up of this agent revealed an increase in survival of patients and a reduction in coagulation time and blood loss. Another research showed that two chitosan formulations could effectively stop the bleeding in a similar liver injury model compared to the control, although no follow-up data were presented [41].

The collateral harm incurred by surrounding organs due to adhesion formation caused by the product is a serious issue in all of these studies. Our study showed that the simple gauze packing had less adhesion and better controlled bleeding when compared to MSD or MSD + surgicel. We observed no adhesions at the site of liver injury in the gauze packing group which meand the efficient pressure of gauze packing provides better coagulation and, as a result, the control of hemorrhage at the site of injury and surgery.

## 5 Limitations and future directions

The current study has some limitations. While this study provides valuable insights into the efficacy of different hemostatic agents in a controlled experimental setting, it is important to note the limitations of extrapolating these results to human clinical practice. In real-life scenarios, particularly in hemodynamically stable patients, angioembolization is often the preferred initial intervention for severe bleeding. However, in cases where such advanced interventions are not immediately available or feasible, rapid initial hemostatic control using available materials is crucial. Thus, the findings from this rat model study should be interpreted with caution, keeping in mind the differences in scale, complexity, and physiological responses between rats and humans. Moreover, although this study focused on XStat minisponge dressing and Surgicel as representative hemostatic agents, we acknowledge that other hemostats may offer enhanced efficacy. Future research should aim to evaluate a wider array of hemostatic materials, including newer and potentially more effective options, to determine the best strategies for managing severe liver injuries.

## 6 Conclusion

In this experimental study comparing gauze packing, XStat minisponge dressings (MDS), and a combination of MDS with Surgicel hemostatic agent for managing grade IV liver injuries in rats, traditional gauze packing emerged as the most effective method for controlling hemorrhage. Gauze packing demonstrated significantly lower blood loss and mortality rates compared to both sponge-based dressings and the combined MDS + Surgicel approach. Furthermore, gauze packing resulted in minimal intraperitoneal adhesions, whereas the other treatments led to adhesions involving the liver, bowel, omentum, and abdominal wall. These findings underscore the continued efficacy of gauze packing in the management of severe liver trauma, highlighting its role as a reliable standard in hemorrhage control.

## CRediT authorship contribution statement

**Mohammad Mehdi Lashkarizadeh:** Writing – review & editing, Methodology, Data curation, Conceptualization. **Arshin Ghaedi:** Writing – review & editing, Writing – original draft, Supervision, Project administration, Conceptualization. **Hojat Abolghasemi:** Methodology, Formal analysis, Data curation. **Mina Rabiee:** Software, Formal analysis, Data curation. **Davood Mehrabani:** Software, Formal analysis, Data curation. **Samad Ahadian:** Methodology, Formal analysis. **Aida Bazrgar:** Writing – review & editing, Writing – original draft. **Shaqayeq Moqbel Esfahani:** Writing – review & editing, Writing – original draft. **Shahram Paydar:** Supervision, Project administration, Funding acquisition, Conceptualization.

## Ethics statement

This study conforms to the Declaration of Helsinki regarding research involving animal subjects and is approved by the Ethics Committee of Shiraz University of Medical Sciences, Shiraz, Iran (IR.SUMS.MED.REC.1398.143).

## Availability of data and materials

The dataset analyzed during the current study are available from the corresponding author on reasonable request.

## Declaration of Generative AI and AI-assisted technologies in the writing process

During the preparation of this work the author(s) used ChatGPT-3.5 (OpenAI) in order to improve readability and language. After using this tool/service, the author(s) reviewed and edited the content as needed and take(s) full responsibility for the content of the publication.

## Funding/financial support

This work was supported by grants from 10.13039/501100004320Shiraz University of Medical Sciences, Shiraz, Iran and was a part of Dr. Mohammad Mehdi Lashkarizadeh thesis.

## Declaration of competing interest

The authors declare the following financial interests/personal relationships which may be considered as potential competing interests: Shahram Paydar reports financial support was provided by 10.13039/501100004320Shiraz University of Medical Sciences. If there are other authors, they declare that they have no known competing financial interests or personal relationships that could have appeared to influence the work reported in this paper.

## References

[1] (2022). National trauma Institute november. https://www.nattrauma.org/trauma-statistics-facts/.

[2] Ferrah N., Cameron P., Gabbe B., Fitzgerald M., Martin K., Beck B. (2019). Trends in the nature and management of serious abdominal trauma. World J. Surg..

[3] Ntundu S.H., Herman A.M., Kishe A., Babu H., Jahanpour O.F., Msuya D. (2019). Patterns and outcomes of patients with abdominal trauma on operative management from northern Tanzania: a prospective single centre observational study. BMC Surg..

[4] El-Menyar A., Abdelrahman H., Al-Hassani A., Peralta R., AbdelAziz H., Latifi R. (2017). Single versus multiple solid organ injuries following blunt abdominal trauma. World J. Surg..

[5] Larsen J.W., Søreide K., Søreide J.A., Tjosevik K., Kvaløy J.T., Thorsen K. (2022). Epidemiology of abdominal trauma: an age-and sex-adjusted incidence analysis with mortality patterns. Injury.

[6] Stagnitti F. (2013). Uncontrolled bleeding in patients with major abdominal trauma. Ann. Ital. Chir..

[7] Li M., Yu W.-K., Wang X.-B., Ji W., Li J.-S., Li N. (2014). Non-operative management of isolated liver trauma. Hepatobiliary Pancreat. Dis. Int..

[8] Chapman W.C., Clavien P.-A., Fung J., Khanna A., Bonham A. (2000). Effective control of hepatic bleeding with a novel collagen-based composite combined with autologous plasma: results of a randomized controlled trial. Arch. Surg..

[9] Heaton N. (2005). Advances and methods in liver surgery: haemostasis. Eur. J. Gastroenterol. Hepatol..

[10] Lisman T., Leebeek F.W. (2007). Hemostatic alterations in liver disease: a review on pathophysiology, clinical consequences, and treatment. Dig. Surg..

[11] Nouri S., Sharif M.R., Sahba S. (2015). The effect of ferric chloride on superficial bleeding. Trauma Mon..

[12] McBee W.L., Koerner K.R. (2005). Review of hemostatic agents used in dentistry. Dent. Today.

[13] Sauaia A., Moore F.A., Moore E.E., Moser K.S., Brennan R., Read R.A. (1995). Epidemiology of trauma deaths: a reassessment. J. Trauma Acute Care Surg..

[14] Hendriks H., Van der Meer J., de Wolf J.T.M., Peeters P., Porte R., De Jong K. (2004). Intraoperative blood transfusion requirement is the main determinant of early surgical re‐intervention after orthotopic liver transplantation. Transpl. Int..

[15] De Boer M.T., Molenaar I.Q., Hendriks H.G., Slooff M.J., Porte R.J. (2005). Minimizing blood loss in liver transplantation: progress through research and evolution of techniques. Dig. Surg..

[16] Goker H., Haznedaroglu I.C., Ercetin S., Kirazli S., Akman U., Ozturk Y. (2008). Haemostatic actions of the folkloric medicinal plant extract Ankaferd Blood Stopper. J. Int. Med. Res..

[17] Nouri S., Sharif M.R., Hosseinpour M., Ehteram H. (2015). The hemostatic effect of aluminum sulfate in liver bleeding in rat. KAUMS Journal (FEYZ)..

[18] Çinar Ç., Odabaş M.E., Akca G., Işik B. (2012). Antibacterial effect of a new haemostatic agent on oral microorganisms. Journal of Clinical and Experimental Dentistry.

[19] Peng H.T. (2020). Hemostatic agents for prehospital hemorrhage control: a narrative review. Mil Med Res.

[20] Kozar R.A., Feliciano D.V., Moore E.E., Moore F.A., Cocanour C.S., West M.A. (2011). Western Trauma Association/critical decisions in trauma: operative management of adult blunt hepatic trauma. J. Trauma.

[21] Gregory K.W., Baranowski L.L., Kalyanpur A., Vine S., Blackwell G., Margolis B. (2014).

[22] Pereira B.M., Bortoto J.B., Fraga G.P. (2018). Topical hemostatic agents in surgery: review and prospects. Rev. Col. Bras. Cir..

[23] Cheng F., Xu L., Dai J., Yi X., He J., Li H.N. (2022). O-carboxymethyl chitosan/oxidized cellulose composite sponge containing ε-poly-l-lysine as a potential wound dressing for the prevention and treatment of postoperative adhesion. Int. J. Biol. Macromol..

[24] Cheng F., Yi X., Dai J., Fan Z., He J., Huang Y. (2023). Photothermal MXene@ Zn-MOF-decorated bacterial cellulose-based hydrogel wound dressing for infectious wound healing. Cell Reports Physical Science.

[25] Li H., Cheng F., Wei X., Yi X., Tang S., Wang Z. (2021). Injectable, self-healing, antibacterial, and hemostatic N, O-carboxymethyl chitosan/oxidized chondroitin sulfate composite hydrogel for wound dressing. Mater. Sci. Eng. C.

[26] Mueller G.R., Pineda T.J., Xie H.X., Teach J.S., Barofsky A.D., Schmid J.R. (2012). A novel sponge-based wound stasis dressing to treat lethal noncompressible hemorrhage. J. Trauma Acute Care Surg..

[27] Rodriguez M.I., Jensen J.T., Gregory K., Bullard M., Longo P., Heidel J. (2017). A novel tamponade agent for management of post partum hemorrhage: adaptation of the Xstat mini-sponge applicator for obstetric use. BMC Pregnancy Childbirth.

[28] Al-Attar N., de Jonge E., Kocharian R., Ilie B., Barnett E., Berrevoet F. (2023). Safety and hemostatic effectiveness of SURGICEL® powder in mild and moderate intraoperative bleeding. Clin. Appl. Thromb. Hemost..

[29] Khoshmohabat H., Paydar S., Makarem A., Karami M.Y., Dastgheib N., Zahraei S.A.H. (2019). A review of the application of cellulose hemostatic agent on trauma injuries. Open Access Emerg. Med..

[30] Cannon J.W. (2018). Hemorrhagic shock. N. Engl. J. Med..

[31] Schoeneberg C., Schilling M., Hussmann B., Schmitz D., Lendemans S., Ruchholtz S. (2017). Preventable and potentially preventable deaths in severely injured patients: a retrospective analysis including patterns of errors. Eur. J. Trauma Emerg. Surg..

[32] Mueller G.R., Pineda T.J., Xie H.X., Teach J.S., Barofsky A.D., Schmid J.R. (2012). A novel sponge-based wound stasis dressing to treat lethal noncompressible hemorrhage. J. Trauma Acute Care Surg..

[33] Romano F., Garancini M., Uggeri F., Degrate L., Nespoli L., Gianotti L. (2012). Bleeding in hepatic surgery: sorting through methods to prevent it. HPB Surg..

[34] Kheirabadi B.S., Mace J.E., Terrazas I.B., Fedyk C.G., Estep J.S., Dubick M.A. (2010). Safety evaluation of new hemostatic agents, smectite granules, and kaolin-coated gauze in a vascular injury wound model in swine. J. Trauma Acute Care Surg..

[35] Alam H.B., Chen Z., Jaskille A., Querol R.I.L.C., Koustova E., Inocencio R. (2004). Application of a zeolite hemostatic agent achieves 100% survival in a lethal model of complex groin injury in swine. J. Trauma Acute Care Surg..

[36] Pusateri A.E., Holcomb J.B., Kheirabadi B.S., Alam H.B., Wade C.E., Ryan K.L. (2006). Making sense of the preclinical literature on advanced hemostatic products. J. Trauma Acute Care Surg..

[37] Xia Y., Yang R., Wang H., Li Y., Fu C. (2022). Application of chitosan-based materials in surgical or postoperative hemostasis. Frontiers in Materials.

[38] Gheorghiță D., Moldovan H., Robu A., Bița A.-I., Grosu E., Antoniac A. (2023). Chitosan-based biomaterials for hemostatic applications: a review of recent advances. Int. J. Mol. Sci..

[39] Zhong Y., Hu H., Min N., Wei Y., Li X., Li X. (2021). Application and outlook of topical hemostatic materials: a narrative review. Ann. Transl. Med..

[40] Hu Z., Zhang D.Y., Lu S.T., Li P.W., Li S.D. (2018). Chitosan-based composite materials for prospective hemostatic applications. Mar. Drugs.

[41] Millner R., Lockhart A.S., Marr R. (2010). Chitosan arrests bleeding in major hepatic injuries with clotting dysfunction: an in vivo experimental study in a model of hepatic injury in the presence of moderate systemic heparinisation. Ann. R. Coll. Surg. Engl..

[42] Bochicchio G.V., Kilbourne M.J., Keledjian K., Hess J., Scalea T. (2010). Evaluation of a new hemostatic agent in a porcine grade V liver injury model. Am. Surg..

